# Generation of efficient mutants of endoglycosidase from *Streptococcus pyogenes* and their application in a novel one-pot transglycosylation reaction for antibody modification

**DOI:** 10.1371/journal.pone.0193534

**Published:** 2018-02-23

**Authors:** Mitsuhiro Iwamoto, Yukiko Sekiguchi, Kensuke Nakamura, Yoshirou Kawaguchi, Takeshi Honda, Jun Hasegawa

**Affiliations:** 1 Modality Research Laboratories, Biologics Division, Daiichi Sankyo Co., Ltd., Tokyo, Japan; 2 Medicinal Chemistry Management Group, Research Function Division, Daiichi Sankyo Co., Ltd., Tokyo, Japan; Weizmann Institute of Science, ISRAEL

## Abstract

The fine structures of Fc *N*-glycan modulate the biological functions and physicochemical properties of antibodies. By remodeling *N*-glycan to obtain a homogeneous glycoform or chemically modified glycan, antibody characteristics can be controlled or modified. Such remodeling can be achieved by transglycosylation reactions using a mutant of endoglycosidase from *Streptococcus pyogenes* (Endo-S) and glycan oxazoline. In this study, we generated improved mutants of Endo-S by introducing additional mutations to the D233Q mutant. Notably, Endo-S D233Q/Q303L, D233Q/E350Q, and several other mutations resulted in transglycosylation efficiencies exceeding 90%, with a single-digit donor-to-substrate ratio of five, and D233Q/Y402F/D405A and several other mutations resulted in slightly reduced transglycosylation efficiencies accompanied by no detectable hydrolysis activity for 48 h. We further demonstrated that the combined use of mutants of Endo-S with Endo-M or Endo-CC, endoglycosidases from *Mucor hiemalis* and *Coprinopsis cinerea*, enables one-pot transglycosylation from sialoglycopeptide to antibodies. This novel reaction enables glycosylation remodeling of antibodies, without the chemical synthesis of oxazoline in advance or *in situ*.

## Introduction

Antibodies are major components of humoral immunity that protect the host from infection by specifically binding to pathogens. IgG, the most common antibody found in the circulation, contributes to host defense via several mechanisms, including complement-dependent cytotoxicity [1–5] and antibody-dependent cell-mediated cytotoxicity [6–9], and the extent of these contributions are significantly influenced by the glycoform of the conserved *N*-linked glycan in the Fc region. In an effort to improve the efficacy and safety of recombinant monoclonal antibody therapeutics, various expression systems have been genetically engineered to produce antibodies with desired glycoforms [10–12]. These systems would also be useful in the production of site-specific antibody–drug conjugates based on glyco-conjugation, which requires a homogeneous glycan for chemical modification [13].

*In vitro* chemoenzymatic remodeling is a promising method that enables high uniformity and precise control over the glycoform by the transfer of oligosaccharide oxazolines using endo-β-*N*-acetylglucosaminidase (ENGase) [14,15]. Endo-S, an ENGase from *Streptococcus pyogenes* that selectively hydrolyzes the chitobiose core of the *N*-linked complex-type glycan of IgG, has been engineered [16–18] and used for the production of antibodies with homogeneous glycoforms to study effects on biological activity [19–21]. However, the large-scale application of this method is hampered by the need for highly active oxazoline at high quantities relative to the antibody owing to the residual hydrolysis activity of the enzyme toward the reaction product.

In this paper, we describe improved mutants of Endo-S that enable efficient transglycosylation using a reduced concentration of oxazoline; the mutants were obtained by site-directed mutagenesis based on structural information and methods adapted from studies of various ENGases [17,22–26]. Further, we describe a novel one-pot reaction to transfer the glycan moiety of sialoglycopeptide (SGP) to antibodies using combinations of mutants of Endo-S and auxiliary ENGase—the mutant of Endo-M from *Mucor hiemalis* [24] or Endo-CC from *Coprinopsis cinerea* [27]. Auxiliary ENGases show activities toward complex-type glycans, but not toward core-fucosylated glycans, unlike Endo-S. Using a combination of ENGases with varying specificities, antibody glycosylation was successfully remodeled without the chemical synthesis of oxazoline.

## Results

### Design and generation of Endo-S mutants

Huang and coworkers have previously identified the Endo-S D233Q and E235A mutants by targeting residues responsible for promoting oxazolinium ion formation by a substrate-assisted mechanism [17]. Additionally, Parson and coworkers independently identified mutations at Q303 and Y305 that assist substrate-binding, but did not find an improved mutant [28]. We targeted a slightly broader region, including other possible residues that may assist substrate-binding or acceptor-binding with substitutions that alter size, charge, or hydrophobicity based on a sequence alignment and the crystal structure of the Endo-S reported by Trastoy and coworkers [29]. Importantly, we introduced most mutations in addition to the D233Q mutation in a successive manner, producing over 120 mutants, including triple and quadruple mutants (S1 Table). The bacterial expression of the mutants yielded comparable protein levels to that of wild-type Endo-S under the same expression and purification conditions.

### Hydrolysis and transglycosylation activity of Endo-S mutants

**Fig 1:**
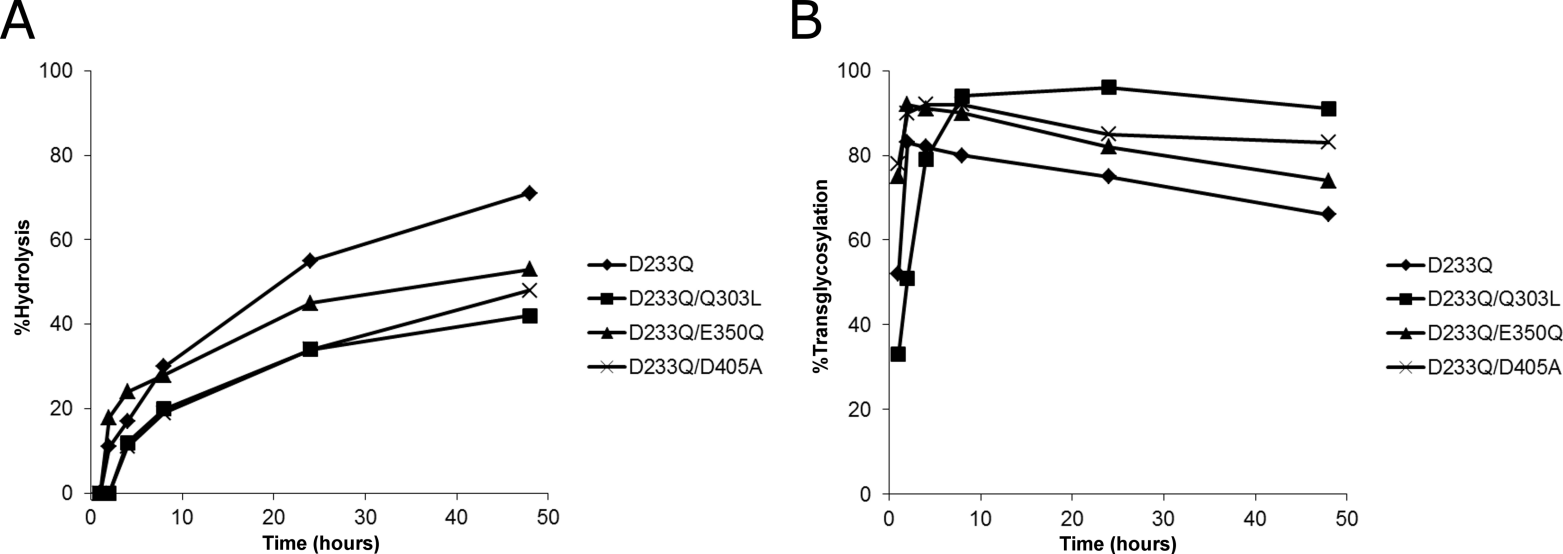
Hydrolysis and transglycosylation reactions using SG-oxazoline as donor substrates by Endo-S mutants. (A) Hydrolysis reactions were performed using trastuzumab and Endo-S mutants at a 50:1 weight ratio. (B) Transglycosylation reactions were carried out using deglycosylated trastuzumab as the acceptor and 5 molar eq of donor oxazoline.

**Fig 2:**
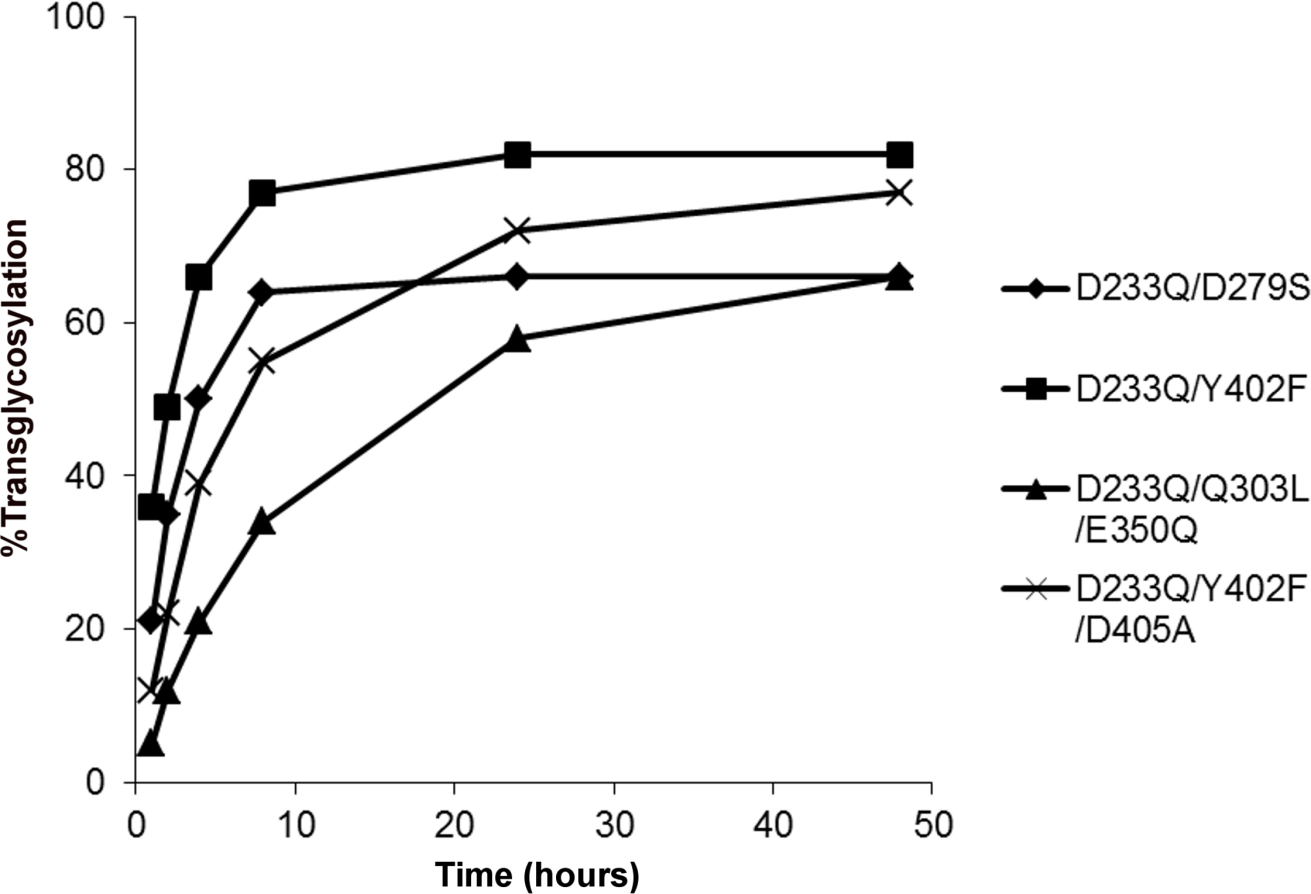
Transglycosylation reactions by Endo-S variants with significantly reduced or no detectable hydrolysis activity. Reactions were carried out using deglycosylated trastuzumab as the acceptor and 5 molar eq of donor oxazoline.

While none of the single mutants of Endo-S displayed improvements over the D233Q mutant, a number of multiple mutants exhibited a reduced hydrolysis efficiency, while retaining a comparable or improved transglycosylation efficiency (S2 Table). The Q303L, E350Q, or D405A mutation combined with D233Q resulted in a slight reduction in hydrolysis efficiency (Fig 1A) and improved maximum transglycosylation efficiency (Fig 1B). Notably, the D233Q/Q303L mutant achieved 96% efficiency at 24 h, with a much slower product hydrolysis phase compared to that of the D233Q mutant (Fig 1B). The D233Q/D405A mutant showed a faster transglycosylation rate, reaching 78% at 2 h, and lower hydrolysis and higher transglycosylation at all time points compared to those of the D233Q mutant. Other mutants, such as D233Q/D279S, D233Q/Y402F, and D233Q/Q303L/E350Q, displayed detectable hydrolysis activity of less than 20% only at 48 h, while retaining a transglycosylation efficiency (Fig 2). Some mutants, including D233Q/Y402F/D405A, did not show detectable hydrolysis activity up to 48 h, though their maximum transglycosylation efficiency did not reach 80% at 48 h (Fig 2, S2 Table).

### One-pot transglycosylation from SGP to trastuzumab using variants of Endo-S and Endo-M

**Fig 3:**
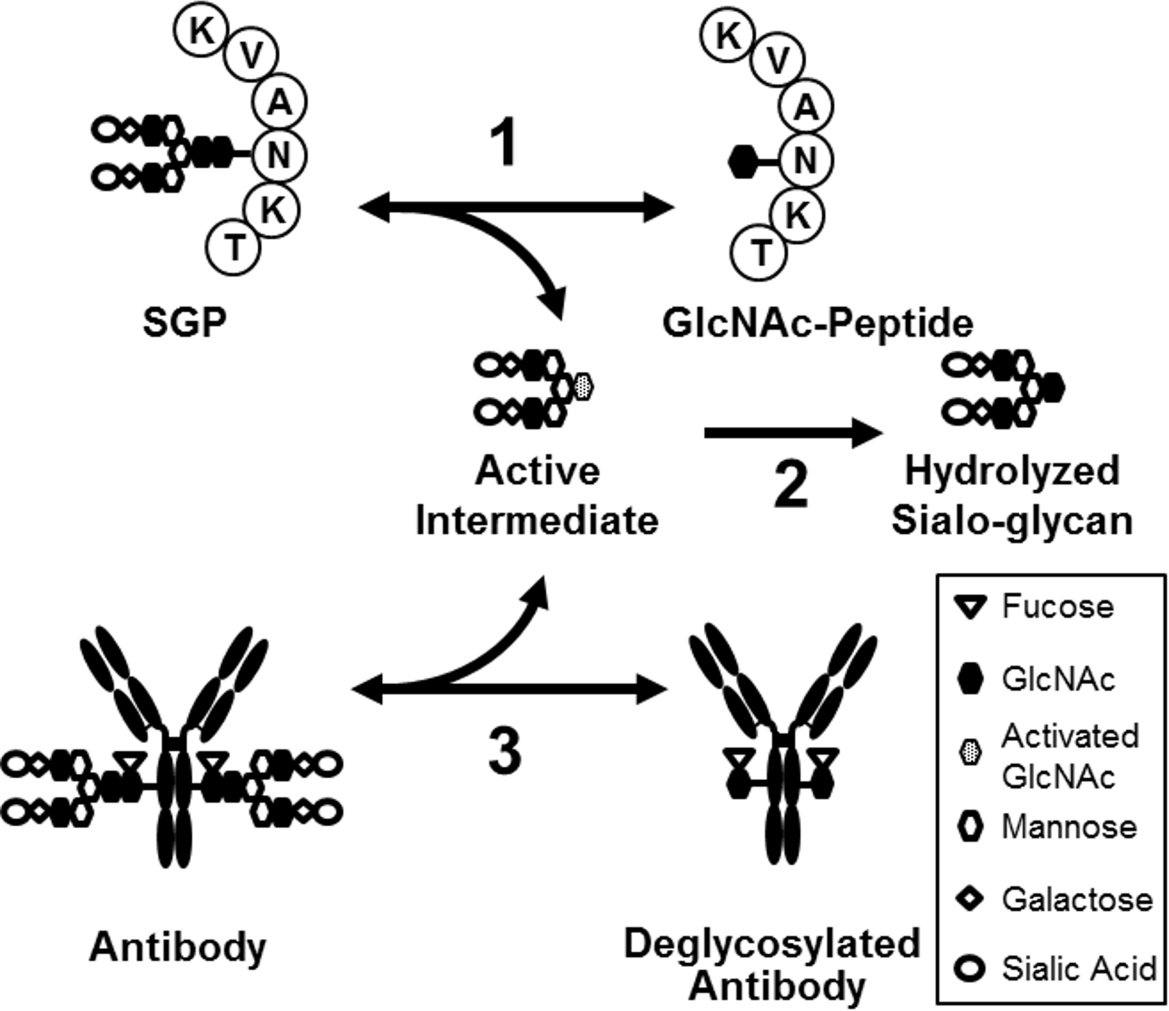
Hypothetical model of the one-pot transglycosylation using SGP as the donor substrate. SGP is reversibly converted to the active intermediate and GlcNAc-peptide mainly by Endo-M (1). The intermediate can be irreversibly hydrolyzed by rapidly by hydrolytic variants of Endo-M and Endo-S or slowly without them (2). If hydrolysis (2) is negligibly slow, the intermediate may be made available for conjugation to the antibody (3) by Endo-S.

To circumvent the need to chemically synthesize sugar oxazoline in advance, we tested a novel one-pot transglycosylation reaction to transfer the glycan moiety from SGP to the antibody using a combination of endoglycosidase mutants with varying target specificities, *i*.*e*., an Endo-S mutant that preferentially targets Fc *N*-glycan and an Endo-M mutant that targets SGP, but not core-fucosylated Fc *N*-glycan. We hypothesized that the active intermediate required for transglycosylation catalyzed by the Endo-S mutant could be prepared *in situ* by an auxiliary endoglycosidase (Fig 3).

**Fig 4:**
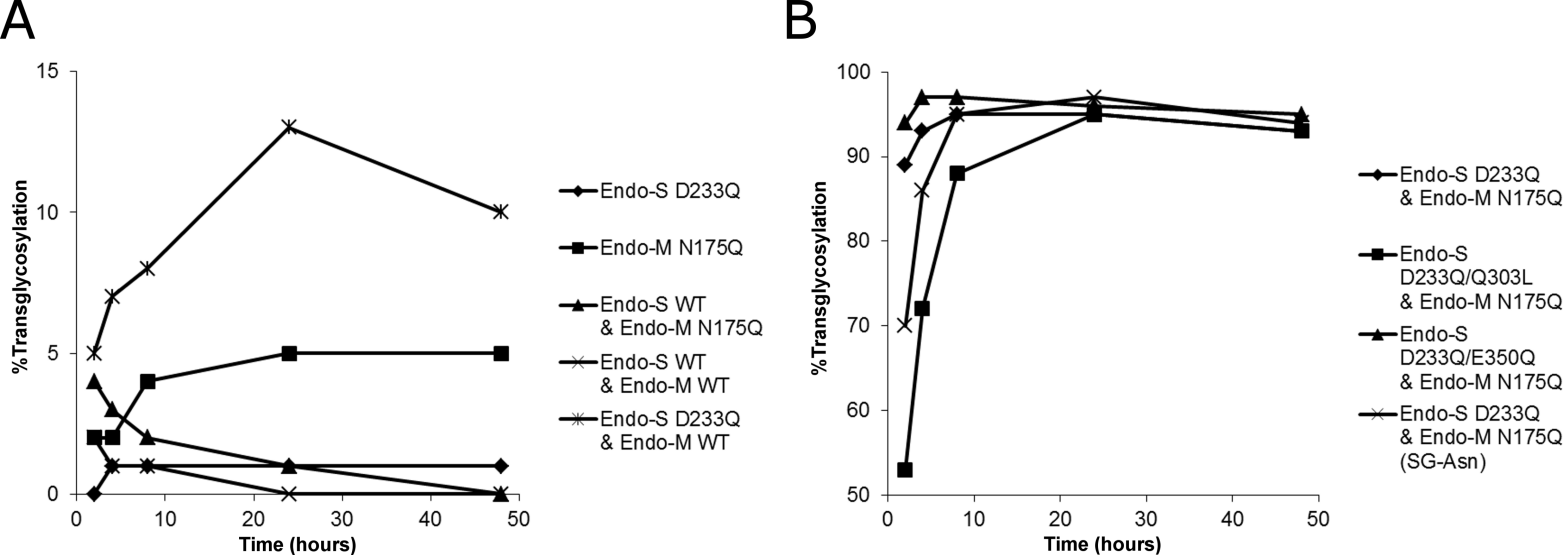
Transglycosylation reactions by variants of Endo-S, Endo-M, and their combinations. (A) Transglycosylation using a mutant alone or a wild-type paired with either a wild-type or a mutant enzyme. (B) Transglycosylation using a pair of mutants. Reactions were carried out using deglycosylated trastuzumab as the acceptor. SGP or SG-Asn was used as the donor substrate at 300 molar eq.

Several combinations of enzyme mutants indeed worked in concert to transfer the glycan moiety of SGP (Tokyo Chemical Industry Co., Ltd., Tokyo, Japan) (Fig 4). While the transglycosylation efficiency of the Endo-S D233Q mutant alone or in combination with wild-type Endo-M reached only 13% (Fig 4A), that of the combination with the Endo-M N175Q mutant reached 95% (Fig 4B). The use of a variant of SGP where the peptide portion was trimmed down to an asparagine residue (SG-Asn) as the donor substrate slightly decreased the reaction rate, but increased the maximum efficiency slightly to 97%. The Endo-S D233Q/E350Q mutant in place of the D233Q mutant could also improve the maximum efficiency to 97%, while wild-type Endo-S severely diminished the transglycosylation activity (Fig 4A).

**Fig 5:**
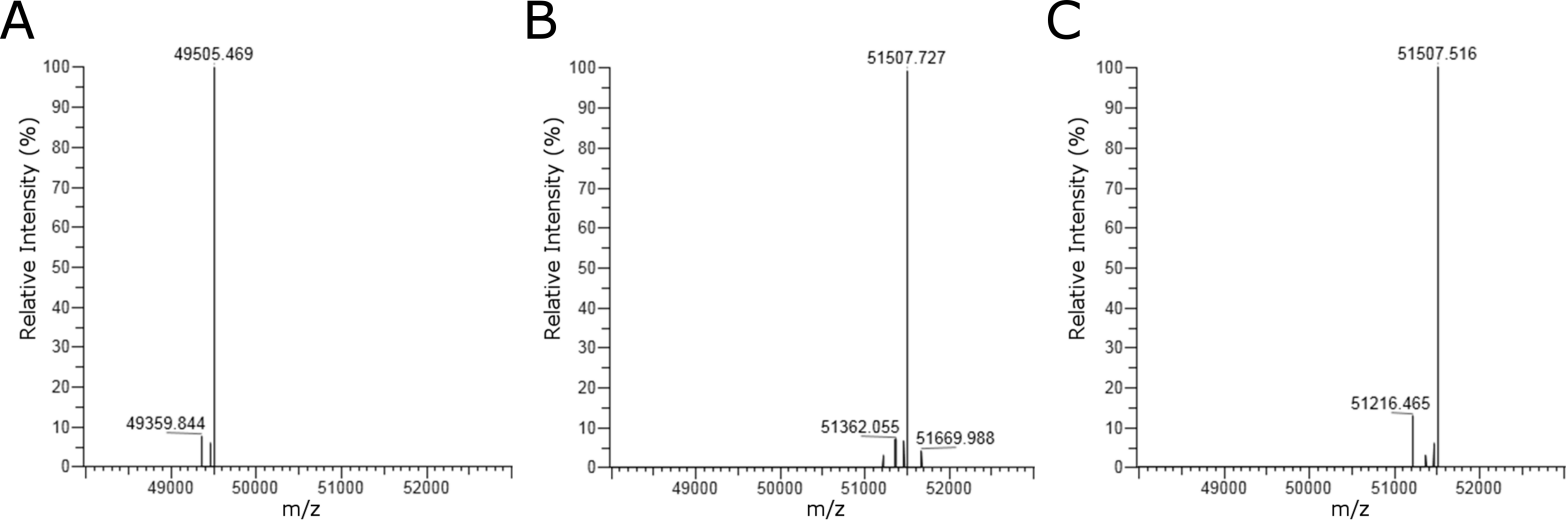
LC-ESI-MS analysis. ESI-MS (after deconvolution) of the heavy chain of deglycosylated trastuzumab (A) and one-pot transglycosylated trastuzumab using SGP (B) or SG-Asn (C) as the donor substrate.

Based on an analysis of the SGP concentration dependency, greater than approximately 100 eq of SGP is required to achieve 90% transglycosylation with the Endo-S D233Q mutant and Endo-M N175Q mutant (S3 Table). Using the combination of Endo-S D233Q and Endo-M N175Q, 10 mg of deglycosylated trastuzumab was one-pot transglycosylated using SGP or SG-Asn as a donor substrate and further purified through a protein A column and CHT column, yielding 8.0 mg of glycosylated-trastuzumab with a homogeneous glycoform, as observed by LC-ESI-MS, in both cases (Fig 5).

### One-pot transglycosylation from SGP to trastuzumab using mutants of Endo-S and Endo-CC

**Fig 6:**
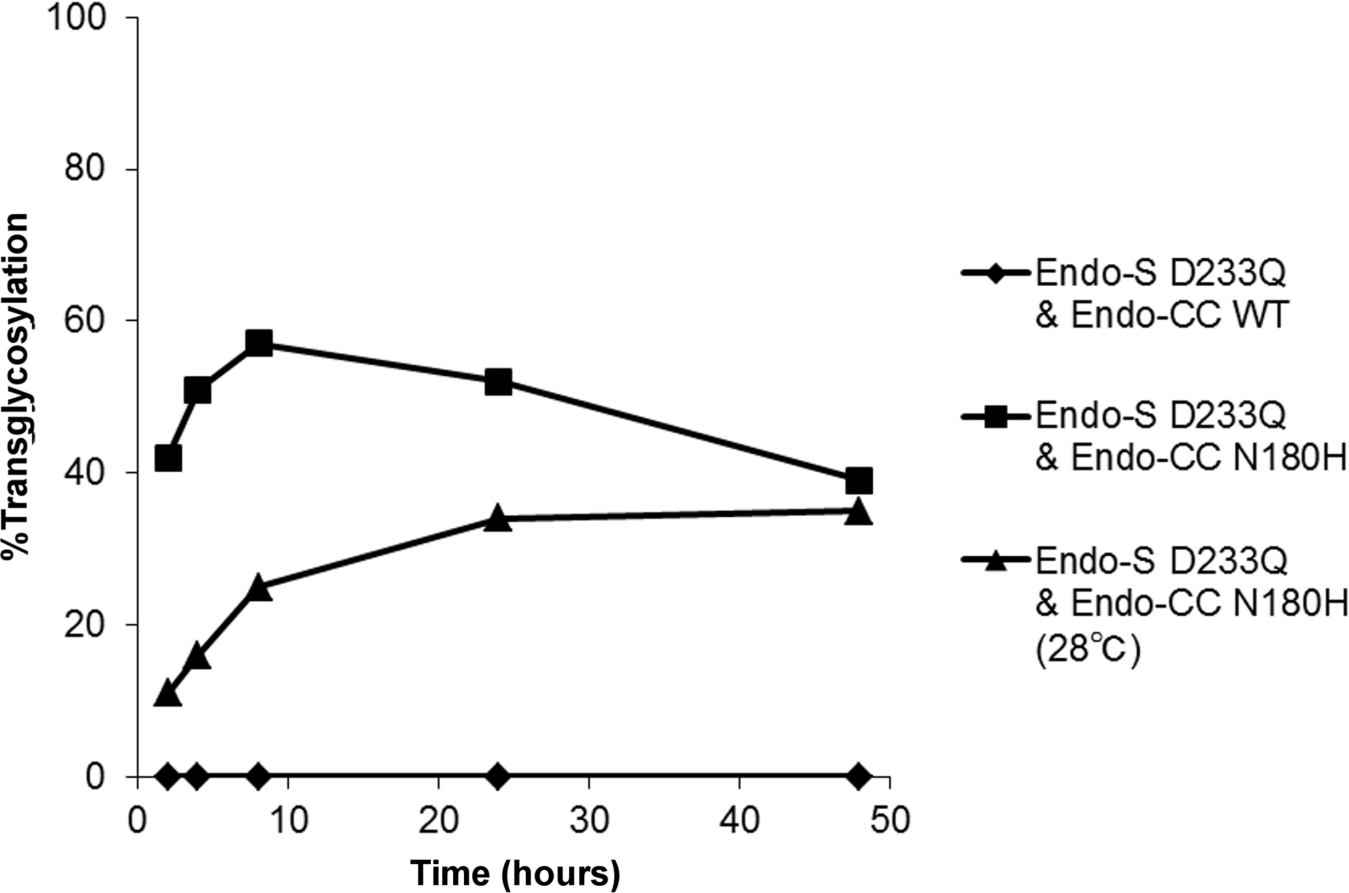
Transglycosylation reactions by pairs of Endo-S and Endo-CC variants. Reactions were carried out using deglycosylated trastuzumab as the acceptor at 37°C or 28°C with 300 molar eq of donor oxazoline.

To test whether Endo-M N175Q could be replaced with another ENGase, Endo-S D233Q was used in combination with variants of Endo-CC at 37°C, the optimal temperature for the latter enzyme. While the reaction with wild-type Endo-CC did not generate a detectable amount of transglycosylation product, that with the Endo-CC N180H mutant showed 57% transglycosylation efficiency at 8 h, but this was followed by significant hydrolysis of the product (Fig 6). Lowering the reaction temperature to 28°C prevented the rapid hydrolysis of the product, but did not improve the maximum efficiency.

## Discussion

In this study, we generated mutants of Endo-S, an endoglycosidase from *Streptococcus pyogenes*, with improved transglycosylation activity compared to that of the previously reported D233Q mutant. Several notable double- and triple-mutants with high transglycosylation activity and low hydrolysis activity were identified. Notably, Endo-S D233Q/Q303L, D233Q/E350Q, and D233Q/D405A demonstrated transglycosylation efficiencies reaching over 90% at a substantially reduced donor-to-acceptor ratio of 5 molar eq (*i*.*e*., 2.5 molar oxazoline per monomeric Fc domain). This is lower than the 20 molar eq used in the recently reported highly efficient D184M mutant of Endo-S2 from *Streptococcus pyogenes* serotype M49 [30]. Endo-S D233Q/E350Q and D233Q/D405A also had higher transglycosylation reaction rates than that of Endo-S D233Q [17]. While several mutants displayed no hydrolytic activity, their transglycosylation efficiency did not show continuous growth after 24 h, likely because of the instability of highly reactive oxazoline at pH 7.5.

**Fig 7:**
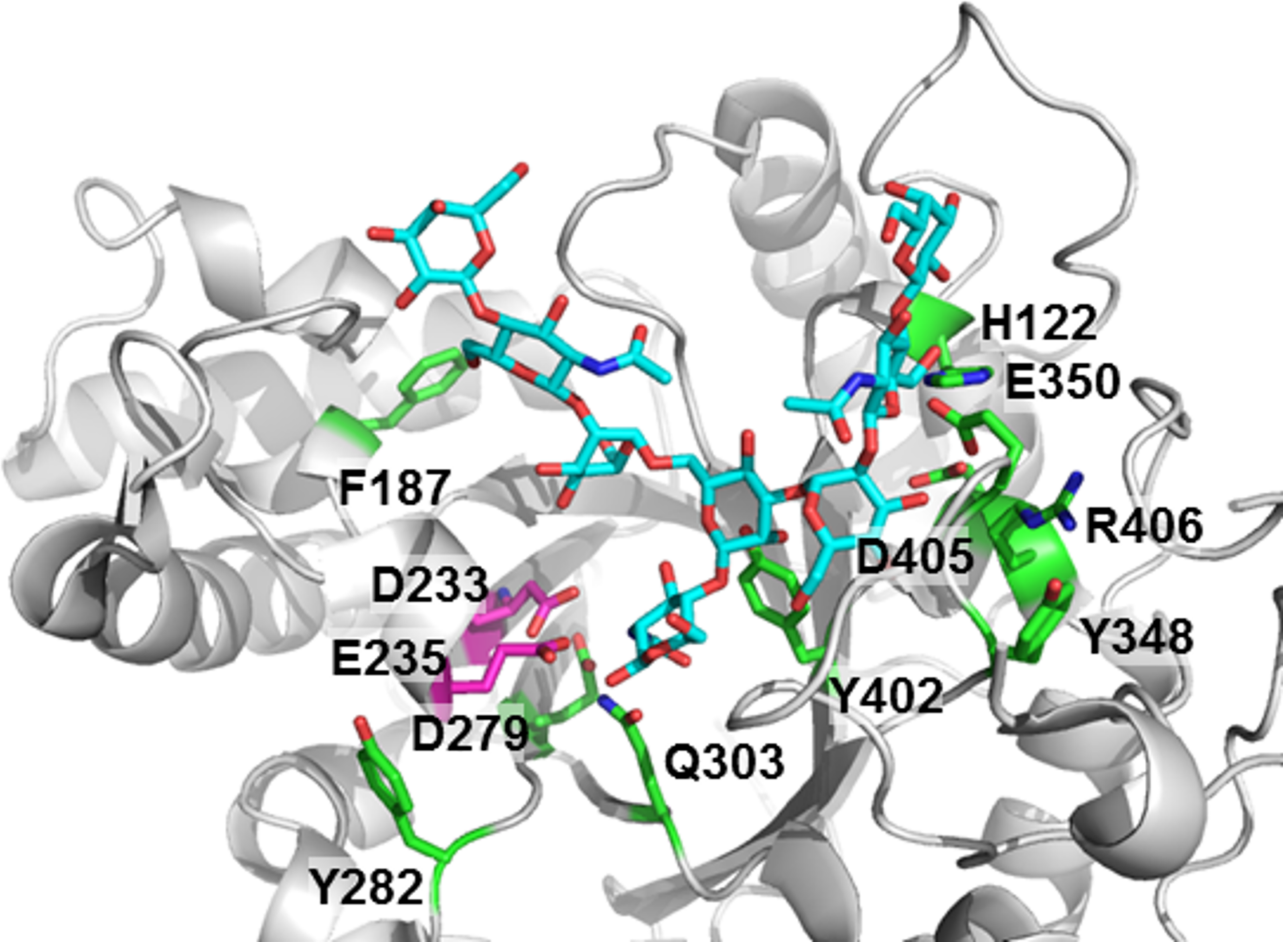
Mutated sites that contributed to improved activity. The catalytic domain of Endo-S (Protein Data Bank code 4NUY, gray), and complex glycan (cyan) from its complex with Endo-F3 (Protein Data Bank code 1EOM, not shown) were approximately placed by superimposing the two catalytic domains. The side chains of key catalytic residues (magenta) and mutated sites that contributed to improved activity (green) are shown in stick form.

In the process of generating efficient mutants, various sites that contributed to the reduced hydrolysis activity were identified, including D279 and Q303 in the proximity of catalytic residues (Fig 7). Most of the other mutations likely alter donor-binding, *i*.*e*., Y402 is in the proximity of the branched mannose residue, H122 and E350 are likely in contact with the α1–3 arm of the substrate glycan, and D405, R406, and Y348 are buried in the interior with respect to the two residues. Only the F187A mutation, which resulted in a very minor improvement, was identified in the proximity of the α1–6 arm. This area was not thoroughly explored, but it is possible that the interaction with the α1–6 arm is not as extensive as interactions with other regions of the glycan.

**Fig 8:**
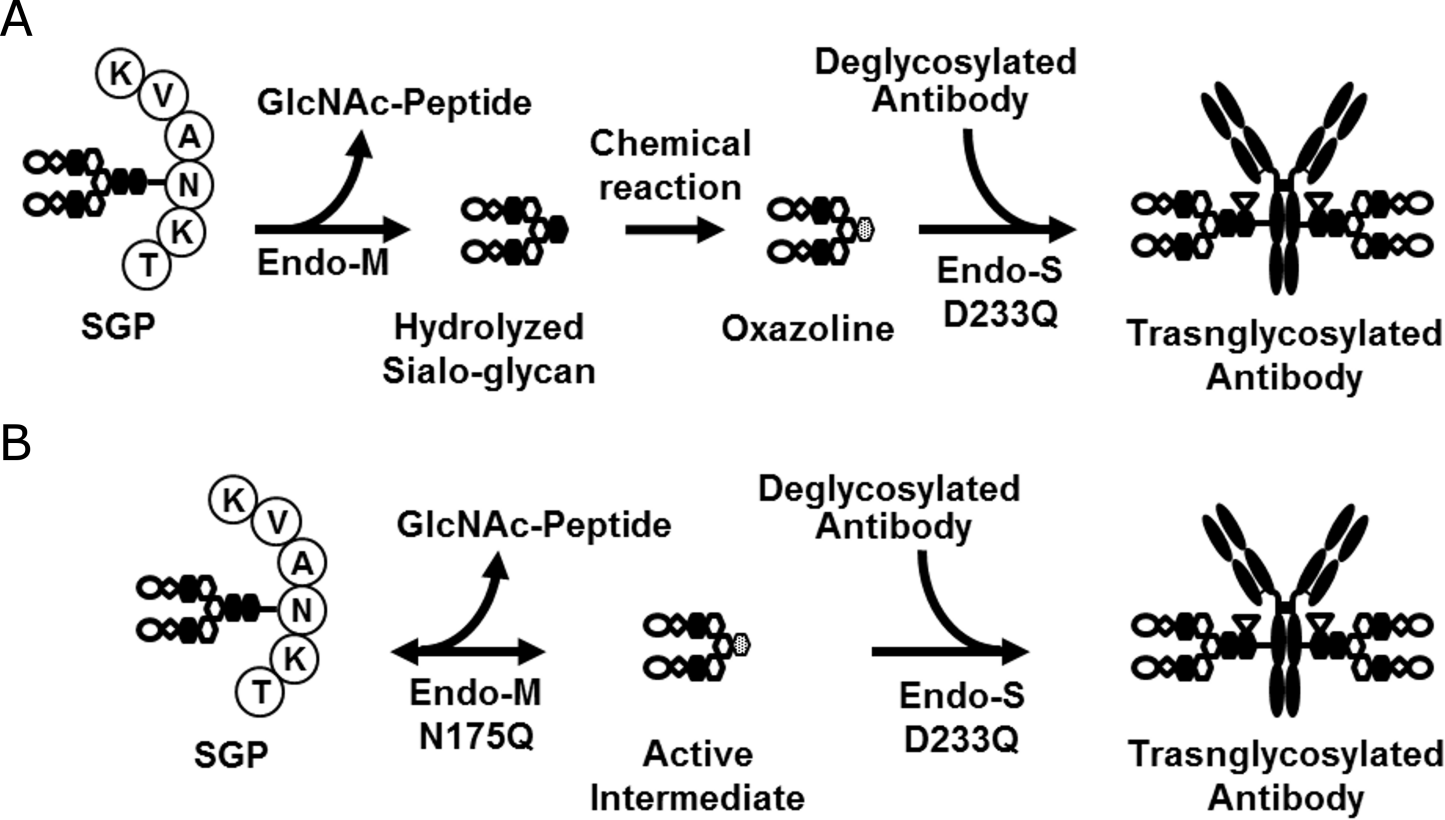
Schematic representation of one-pot transglycosylation reactions. (A) Scheme proposed by Li et al. Wild-type Endo-M efficiently hydrolyzes SGP. Hydrolyzed sialoglycan is subsequently converted to SG-oxazoline *in situ* to provide the active donor substrate for transglycosylation catalyzed by the Endo-S D233Q mutant. (B) Scheme proposed in this study. The Endo-M N175Q mutant converts SGP to the active intermediate, which is then conjugated to the deglycosylated antibody by an Endo-S mutant.

Importantly, we showed that the glycan moiety could be transferred from SGP to the antibody by the combination of ENGase mutants. Initially, the use of wild-type Endo-S and Endo-M yielded unsuccessful results due to the hydrolysis of the product and active intermediate, respectively. However, use of the Endo-S D233Q/E350 mutant with the Endo-M N175Q mutant to minimize hydrolysis greatly improved the maximum transglycosylation efficiency to 97%. Although the detailed process remains unclear, the active intermediate generated by the Endo-M mutant may have been transferred to the Endo-S mutant for subsequent transglycosylation of the antibody. In contrast to the recently reported chemical approach in which sialoglycan hydrolyzed by wild-type Endo-M was chemically converted to SG-oxazoline *in situ* [31] (Fig 8A), our approach with the Endo-M N175Q mutant [23,24] did not require preparation of oxazoline as a glycan donor (Fig 8B).

The applicability of the Endo-CC mutant in place of the Endo-M mutant suggests that this type of reaction can be carried out with different combinations of ENGases and their substrates (Fig 6). Additionally, this reaction may not be limited to rare mutants. The combination of wild-type Endo-S and Endo-M resulted in 1% transglycosylation efficiencies at several time points, despite the predominantly hydrolytic nature of the enzymes, partly because of the high donor-to-acceptor ratio and high transglycosylation activity of Endo-M (Fig 4A).

Unexpectedly, the Endo-M N175Q mutant, without the help of Endo-S, showed approximately 5% transglycosylation efficiency towards core-fucosylated IgG (Fig 4A). This may be in conflict with a previous report by Katoh et al. They reported that the Endo-M N175Q mutant scarcely transglycosylated SG-oxazoline to the core-fucosylated acceptor at a donor-to-acceptor ratio of 1:1, whereas their engineered Endo-M N175Q/W251 double mutant showed approximately 5% transglycosylation efficiency [32]. The apparent conflict may be related to differences in conditions such as the type of donor glycan and its ratio to acceptor. Further studies of ENGases and their one-pot reactions are required to develop more efficient conditions.

## Conclusions

Enzymatic remodeling of glycan has extensive research uses, but is limited for large-scale applications. In this study, over 120 mutants of Endo-S were screened with the aim of obtaining an efficient enzyme with improved transglycosylation activity and reduced hydrolysis activity. By introducing additional mutations into the D233Q mutant [17], we obtained significantly improved Endo-S mutants with transglycosylation efficiencies reaching over 90% at a donor-to-acceptor ratio of five. Using the mutant of Endo-M and Endo-CC [24,27], we also developed a novel one-pot transglycosylation reaction to circumvent the need for the chemical synthesis of a highly active intermediate. Further optimization of the one-pot reaction, especially for combinations of ENGase mutants, is expected to lower the required donor-to-acceptor ratio. These developments represent progress towards large-scale manufacturing using chemoenzymatic processes.

## Materials and methods

### Site-directed mutagenesis and the expression and purification of recombinant Endo-S

The synthesized DNA fragment encoding Endo-S from *Streptococcus pyogenes* was subcloned into the pGEX-4T-3 vector (GE Healthcare, Little Chalfont, UK) for the expression of glutathione-*S*-transferase (GST)-fused Endo-S. Mutations were introduced using the QuikChange II Site-Directed Mutagenesis Kit (Agilent, Santa Clara, CA, USA). The plasmids containing mutated Endo-S genes were transformed into *Escherichia coli* BL21(DE3). The transformants were cultured in 50 mL of Luria Broth supplemented with 100 μg/mL ampicillin, and overexpression was induced by the addition of 0.1 mM isopropyl-β-d-1-thio-galactopyranoside. After 20 h of culture at 16°C, the cells were harvested by centrifugation. The cell pellets were resuspended in PBS (8 g/L NaCl, 2 g/L KCl, 1.15 g/L Na_2_HPO_4_, 0.2 g/L KH_2_PO_4_, pH 7.4, Takara-Bio, Shiga, Japan) containing 0.5 mg/mL lysozyme (Wako, Osaka, Japan) and 40 U of DNaseI (Invitrogen, Carlsbad, CA, USA) and then sonicated. The supernatant of the cell lysate was affinity-purified with Glutathione Sepharose 4B (GE Healthcare), buffer exchanged to PBS, and concentrated to approximately 2 to 7 mg/mL by Amicon Ultrafiltration (10 kDa; Millipore, Burlington, MA, USA). For the large-scale purification of selected Endo-S variants, 1 L of culture was used and affinity-purified materials were gel-filtered in PBS with HiLoad 26/600 Superdex 200 prep grade (GE Healthcare), instead of diafiltration, to remove 10 mM glutathione used for elution from Glutathione Sepharose 4B.

### Deglycosylation of trastuzumab by Endo-S

A solution of trastuzumab (100 mg; Herceptin from Genentech Inc., San Francisco, CA, USA) was incubated with Endo-S (500 μg) at 37°C in a total volume of 4.39 mL of 50 mM phosphate buffer (pH 6.0) for 4 h. The mixture was immediately subjected to affinity chromatography with HiTrap rProteinA HP (GE Healthcare), and hydroxyapatite chromatography with CHT Type I (Bio-Rad, Hercules, CA, USA), and buffer-exchanged to 50 mM Tris-HCl buffer (pH 7.4), yielding 85.7 mg of deglycosylated trastuzumab. LC-MS results for the heavy chain of (Fucα1, 6) GlcNAc-trastuzumab, M = 49506.0 Da; found (m/z), 49505.5 (deconvolution data).

### Enzyme assays

The assay for the hydrolysis activity of each Endo-S mutant (10 μg) was performed at 30°C with commercial trastuzumab (500 μg) as a substrate in Tris-HCl buffer (50 mM, pH 7.4, 30 μL). The transglycosylation activity of each Endo-S mutant (16 μg) was assayed at 30°C with deglycosylated trastuzumab (800 μg) and SG-oxazoline [33] (53.5 μg, 5 eq) in Tris-HCl buffer (50 mM, pH 7.4, 49.07 μL). In both assays, aliquots of the reaction mixture were sampled at 1, 2, 4, 8, 24, and 48 h, and instantly diluted with 4 μL of deionized water and 2 μL of a 29:1 mixture of Experion Pro260 Sample Buffer (Bio-Rad) and NuPAGE Sample Reducing Agent (Invitrogen). The aliquots were briefly heat-denatured, further diluted with 84 μL of deionized water, and analyzed using the Experion Pro260 Analysis Kit on the Experion Automated Electrophoresis Station (Bio-Rad) (S1 Fig). The peak area of the deglycosylated or transglycosylated heavy chain relative to the total heavy chain was calculated using Experion software.

### Assay for the one-pot transglycosylation of deglycosylated trastuzumab by the combined use of ENGases

A solution of deglycosylated trastuzumab (1 mg) and SGP derived from egg yolk (Tokyo Chemical Industry Co., Ltd., Tokyo, Japan, 5.82 mg, 300 eq) were incubated with an Endo-S mutant (20 μg) and/or Endo-M mutant (Tokyo Chemical Industry Co., Ltd., Tokyo, Japan, 5 mU) at 28°C in Tris-HCl buffer (50 mM, pH 7.4, 64.1 μL). The SGP concentration dependency was additionally tested at 50, 100, 200, and 400 eq for the reaction with Endo-S D233Q in concert with Endo-M N175Q. The assay using SG-Asn [28] (4.74 mg, 300 eq) as a substrate was carried out at a slightly reduced volume of 58.7 μL. The assay using an Endo-S mutant (20 μg) and Endo-CC (17 mU) or Endo-CC N180H mutant (33 mU) was carried out at 28°C and/or 37°C in Tris-HCl buffer (50 mM, pH 7.4, 74.1 μL). In all assays, aliquots of the reaction mixture were sampled at 1, 2, 4, 8, 24, and 48 h, and analyzed using the Experion Pro260 Analysis Kit on the Experion Automated Electrophoresis Station (Bio-Rad). The peak area of the product relative to the total heavy chain was calculated using Experion software.

### One-pot transglycosylation of deglycosylated trastuzumab at a medium scale

A solution of deglycosylated trastuzumab (10 mg) and SGP (58.2 mg, 300 eq) was incubated with the Endo-S D233Q mutant (200 μg) and Endo-M N175Q mutant (0.05 U) at 28°C in Tris-HCl buffer (50 mM, pH 7.4, 600 μL) for 4.5 h. The mixture was immediately subjected to affinity chromatography with HiTrap rProteinA FF (GE Healthcare) and hydroxyapatite chromatography with CHT Type I (Bio-Rad), and buffer-exchanged to sodium phosphate buffer (137 mM NaCl, 10 mM Na_2_HPO_4_/NaH_2_PO_4_ pH 6.0, Nacalai Tesque, Kyoto, Japan) supplemented with 5 mM EDTA (Nacalai Tesque, Kyoto, Japan), yielding 8.0 mg of 11.82 mg/mL SG-trastuzumab in 680 μL. LC-MS results for the heavy chain of SG-trastuzumab, M = 51508.7 Da; found (m/z), 51508.0 (deconvolution data). Similarly, a reaction using SG-Asn (47.3 mg, 300 eq) was carried out at slightly reduced volume of 570 μL, yielding 8.0 mg of 10.31 mg/mL SG-trastuzumab in 780 μL. LC-MS: calculated for the heavy chain of SG-trastuzumab, M = 51508.7 Da; found (m/z), 51508.0 (deconvolution data).

## Supporting information

S1 Table Endo-S mutants prepared in this study. Mutants were expressed as GST-fusion proteins in a 50-mL culture volume of BL21(DE3). The final concentration and volume are indicated.
(DOCX)

S2 Table Mutants with improved efficiency. The hydrolysis and transglycosylation efficiencies of Endo-S D233Q and other mutants that displayed reduced hydrolysis or enhanced transglycosylation efficiency at any time point compared to the Endo-S D233Q mutant.
(DOCX)

S3 Table The SGP concentration-dependency in one-pot transglycosylation. The one-pot transglycosylation efficiency by Endo-S D233Q and Endo-M N175Q using various concentrations of SGP as a donor substrate.
(DOCX)

S1 Fig Electrophoretic analysis of the deglycosylation reaction. (A) The electropherogram of the Endo-S D233Q-catalyzed deglycosylation reaction at 1 h obtained using Experion Automated Electrophoresis Station (Bio-Rad). (B) Comparison of the heavy chain peak at various time points of the deglycosylation reaction demonstrates that the relative amount of glycosylated and deglycosylated heavy chains can be approximately quantified.
(TIF)

## References

[1] Kaneko Y, Nimmerjahn F, Ravetch JV. Anti-inflammatory activity of immunoglobulin G resulting from Fc sialylation. Science. 2006;313: 670–673. doi: 10.1126/science.1129594 16888140 10.1126/science.1129594

[2] Anthony RM, Nimmerjahn F, Ashline DJ, Reinhold VN, Paulson JC, Ravetch JV. Recapitulation of IVIG anti-inflammatory activity with a recombinant IgG Fc. Science. 2008;320: 373–376. doi: 10.1126/science.1154315 18420934 10.1126/science.1154315 PMC2409116

[3] Anthony RM, Wermeling F, Karlsson MCI, Ravetch JV. Identification of a receptor required for the anti-inflammatory activity of IVIG. Proc Natl Acad Sci. 2008;105: 19571–19578. doi: 10.1073/pnas.0810163105 19036920 10.1073/pnas.0810163105 PMC2604916

[4] Washburn N, Schwab I, Ortiz D, Lansing JC, Medeiros A, Tyler S, et al Correction for Washburn et al., Controlled tetra-Fc sialylation of IVIg results in a drug candidate with consistent enhanced anti-inflammatory activity. Proc Natl Acad Sci. 2015;112: E4339–E4339. doi: 10.1073/pnas.1512309112 25733881 10.1073/pnas.1422481112 PMC4371931

[5] Schwab I, Mihai S, Seeling M, Kasperkiewicz M, Ludwig RJ, Nimmerjahn F. Broad requirement for terminal sialic acid residues and FcγRIIB for the preventive and therapeutic activity of intravenous immunoglobulins in vivo. Eur J Immunol. 2014;44: 1444–1453. doi: 10.1002/eji.201344230 24505033 10.1002/eji.201344230

[6] Shields RL, Lai J, Keck R, O’Connell LY, Hong K, Gloria Meng Y, et al Lack of fucose on human IgG1 N-linked oligosaccharide improves binding to human FcγRIII and antibody-dependent cellular toxicity. J Biol Chem. 2002;277: 26733–26740. doi: 10.1074/jbc.M202069200 11986321 10.1074/jbc.M202069200

[7] Shinkawa T, Nakamura K, Yamane N, Shoji-Hosaka E, Kanda Y, Sakurada M, et al The absence of fucose but not the presence of galactose or bisecting N-acetylglucosamine of human IgG1 complex-type oligosaccharides shows the critical role of enhancing antibody-dependent cellular cytotoxicity. J Biol Chem. 2003;278: 3466–3473. doi: 10.1074/jbc.M210665200 12427744 10.1074/jbc.M210665200

[8] Okazaki A, Shoji-Hosaka E, Nakamura K, Wakitani M, Uchida K, Kakita S, et al Fucose depletion from human IgG1 oligosaccharide enhances binding enthalpy and association rate between IgG1 and FcγRIIIa. J Mol Biol. 2004;336: 1239–1249. doi: 10.1016/j.jmb.2004.01.007 15037082 10.1016/j.jmb.2004.01.007

[9] Iida S, Misaka H, Inoue M, Shibata M, Nakano R, Yamane-Ohnuki N, et al Nonfucosylated therapeutic IgG1 antibody can evade the inhibitory effect of serum immunoglobulin G on antibody-dependent cellular cytotoxicity through its high binding to FcγRIIIa. Clin Cancer Res. 2006;12: 2879–2887. doi: 10.1158/1078-0432.CCR-05-2619 16675584 10.1158/1078-0432.CCR-05-2619

[10] Yamane-Ohnuki N, Kinoshita S, Inoue-Urakubo M, Kusunoki M, Iida S, Nakano R, et al Establishment of FUT8 knockout Chinese hamster ovary cells: An ideal host cell line for producing completely defucosylated antibodies with enhanced antibody-dependent cellular cytotoxicity. Biotechnol Bioeng. 2004;87: 614–622. doi: 10.1002/bit.20151 15352059 10.1002/bit.20151

[11] Rouwendal GJA, Wuhrer M, Deelder M, Bakker H, Stoopen GM, Hokke H, et al Efficient introduction of a bisecting GlcNAc residue in tobacco N -glycans by expression of the gene encoding human N -acetylglucosaminyltransferase III. 2007;17: 334–344. doi: 10.1093/glycob/cwl078 17179169 10.1093/glycob/cwl078

[12] Ye J, Ly J, Watts K, Hsu A, Walker A, Mclaughlin K, et al Optimization of a glycoengineered *Pichia pastoris* cultivation process for commercial antibody production. Biotechnol Prog. 2011;27: 1744–1750. doi: 10.1002/btpr.695 22002933 10.1002/btpr.695

[13] Agarwal P, Bertozzi CR. Site-specific antibody-drug conjugates: The nexus of bioorthogonal chemistry, protein engineering, and drug development. Bioconjug Chem. 2015;26: 176–192. doi: 10.1021/bc5004982 25494884 10.1021/bc5004982 PMC4335810

[14] Wang L-X, Lomino J V. Emerging technologies for making glycan-defined glycoproteins. ACS Chem Biol. 2012;7: 110–122. doi: 10.1021/cb200429n 22141574 10.1021/cb200429n PMC3262938

[15] Zou G, Ochiai H, Huang W, Yang Q, Li C, Wang L-X. Chemoenzymatic synthesis and Fcγ receptor binding of homogeneous glycoforms of antibody Fc domain. Presence of a bisecting sugar moiety enhances the affinity of Fc to FcγIIIa receptor. J Am Chem Soc. 2011;133: 18975–18991. doi: 10.1021/ja208390n 22004528 10.1021/ja208390n PMC3218234

[16] Allhorn M, Olsén A, Collin M. EndoS from *Streptococcus pyogenes* is hydrolyzed by the cysteine proteinase SpeB and requires glutamic acid 235 and tryptophans for IgG glycan-hydrolyzing activity. BMC Microbiol. 2008;8: 3 https://doi.org/10.1186/1471-2180-8-3. 18182097 10.1186/1471-2180-8-3 PMC2266755

[17] Huang W, Giddens J, Fan S, Christian T, Wang L-X. Chemoenzymatic glycoengineering of intact IgG antibodies for gain of functions. J Am Chem Soc. 2012;134: 12308–12318. doi: 10.1021/ja3051266 22747414 10.1021/ja3051266 PMC3427744

[18] Goodfellow JJ, Baruah K, Yamamoto K, Bonomelli C, Krishna B, Harvey DJ, et al An endoglycosidase with alternative glycan specificity allows broadened glycoprotein remodelling. J Am Chem Soc. 2012;134: 8030–8033. doi: 10.1021/ja301334b 22551167 10.1021/ja301334b

[19] Quast I, Keller CW, Maurer MA, Giddens JP, Tackenberg B, Wang L-X, et al Sialylation of IgG Fc domain impairs complement-dependent cytotoxicity. J Clin Invest. 2015;125: 4160–4170. doi: 10.1172/JCI82695 26436649 10.1172/JCI82695 PMC4639970

[20] Lin C-W, Tsai M-H, Li S-T, Tsai T-I, Chu K-C, Liu Y-C, et al A common glycan structure on immunoglobulin G for enhancement of effector functions. Proc Natl Acad Sci. 2015;112: 10611–10616. doi: 10.1073/pnas.1513456112 26253764 10.1073/pnas.1513456112 PMC4553773

[21] Kurogochi M, Mori M, Osumi K, Tojino M, Sugawara S, Takashima S, et al Glycoengineered monoclonal antibodies with homogeneous glycan (M3, G0, G2, and A2) using a chemoenzymatic approach have different affinities for FcγRIIIa and variable antibody-dependent cellular cytotoxicity activities. PLoS One. 2015;10: e0132848 doi: 10.1371/journal.pone.0132848 26200113 10.1371/journal.pone.0132848 PMC4511734

[22] Huang W, Li C, Li B, Umekawa M, Yamamoto K, Zhang X, et al Glycosynthases enable a highly efficient chemoenzymatic synthesis of N-Glycoproteins carrying intact natural N-Glycans. J Am Chem Soc. 2009;131: 2214–2223. doi: 10.1021/ja8074677 19199609 10.1021/ja8074677 PMC2640449

[23] Umekawa M, Li C, Higashiyama T, Huang W, Ashida H, Yamamoto K, et al Efficient glycosynthase mutant derived from *Mucor hiemalis* Endo-β-*N*-acetylglucosaminidase capable of transferring oligosaccharide from both sugar oxazoline and natural N-glycan. J Biol Chem. 2010;285: 511–521. doi: 10.1074/jbc.M109.059832 19880511 10.1074/jbc.M109.059832 PMC2804199

[24] Umekawa M, Huang W, Li B, Fujita K, Ashida H, Wang L-X, et al Mutants of *Mucor hiemalis* endo-β-*N*-acetylglucosaminidase show enhanced transglycosylation and glycosynthase-like activities. J Biol Chem. 2008;283: 4469–4479. doi: 10.1074/jbc.M707137200 18096701 10.1074/jbc.M707137200

[25] Fan SQ, Huang W, Wang L-X. Remarkable transglycosylation activity of glycosynthase mutants of endo-D, an endo-β-*N*-acetylglucosaminidase from Streptococcus pneumoniae. J Biol Chem. 2012;287: 11272–11281. doi: 10.1074/jbc.M112.340497 22318728 10.1074/jbc.M112.340497 PMC3322811

[26] Giddens JP, Lomino JV, Amin MN, Wang L-X. Endo-F3 glycosynthase mutants enable chemoenzymatic synthesis of core-fucosylated triantennary complex type glycopeptides and glycoproteins. J Biol Chem. 2016;291: 9356–9370. doi: 10.1074/jbc.M116.721597 26966183 10.1074/jbc.M116.721597 PMC4861498

[27] Higuchi Y, Eshima Y, Huang Y, Kinoshita T, Sumiyoshi W, Nakakita S, et al Highly efficient transglycosylation of sialo-complex-type oligosaccharide using *Coprinopsis cinerea* endoglycosidase and sugar oxazoline. Biotechnol Lett. 2017;39: 157–162. doi: 10.1007/s10529-016-2230-0 27714557 10.1007/s10529-016-2230-0

[28] Parsons TB, Struwe WB, Gault J, Yamamoto K, Taylor TA, Raj R, et al Optimal synthetic glycosylation of a therapeutic antibody. Angew Chem Int Ed Engl. 2016;55: 2361–2367. doi: 10.1002/anie.201508723 26756880 10.1002/anie.201508723 PMC4973692

[29] Trastoy B, Lomino JV, Pierce BG, Carter LG, Günther S, Giddens JP, et al Crystal structure of *Streptococcus pyogenes* EndoS, an immunomodulatory endoglycosidase specific for human IgG antibodies. Proc Natl Acad Sci U S A. 2014;111: 6714–6719. doi: 10.1073/pnas.1322908111 24753590 10.1073/pnas.1322908111 PMC4020096

[30] Li T, Tong X, Yang Q, Giddens JP, Wang L-X. Glycosynthase mutants of endoglycosidase S2 show potent transglycosylation activity and remarkably relaxed substrate specificity for antibody glycosylation remodeling. J Biol Chem. 2016;291: 16508–16518. doi: 10.1074/jbc.M116.738765 27288408 10.1074/jbc.M116.738765 PMC4974367

[31] Tang F, Yang Y, Tang Y, Tang S, Yang L, Sun B, et al One-pot N-glycosylation remodeling of IgG with non-natural sialylglycopeptides enables glycosite-specific and dual-payload antibody–drug conjugates. Org Biomol Chem. 2016;14: 9501–9518. doi: 10.1039/c6ob01751g 27714198 10.1039/c6ob01751g

[32] Katoh T, Katayama T, Tomabechi Y, Nishikawa Y, Kumada J, Matsuzaki Y, Yamamoto K. Generation of a mutant mucor hiemalis endoglycosidase that acts on core-fucosylated N-glycans. J Biol Chem. 2016;291: 23305–23317. doi: 10.1074/jbc.M116.737395 27629418 10.1074/jbc.M116.737395 PMC5087746

[33] Noguchi M, Fujieda T, Huang WC, Ishihara M, Kobayashi A, Shoda S. A practical one-step synthesis of 1,2-oxazoline derivatives from unprotected sugars and its application to chemoenzymatic β-*N* -acetylglucosaminidation of disialo-oligosaccharide. Helevetica Chim Acta. 2012;95: 1928–1936. doi: 10.1002/hlca.201200414

