# Supplementary material for: Generation of efficient mutants of endoglycosidase from Streptococcus pyogenes and their application in a novel one-pot transglycosylation reaction for antibody modification
Source: PLoS One. 2018 Feb 23;13(2):e0193534. doi: 10.1371/journal.pone.0193534 (PMC5825150; doi:10.1371/journal.pone.0193534)
Supplement: S1 Table — Mutants were expressed as GST-fusion proteins in a 50-mL culture volume of BL21(DE3). The final concentration and volume are indicated. (DOCX) [file pone.0193534.s001.docx]

| No. | Mutant | Concentration (mg/ml) | Volume (ml) |
| --- | --- | --- | --- |
| 1 | D233N | 3.7 | 1.1 |
| 2 | D233E | 3.9 | 1.1 |
| 3 | D233S | 4.4 | 1.1 |
| 4 | D233C | 4.1 | 1.1 |
| 5 | E235Q | 4.7 | 2.8 |
| 6 | E235D | 4.8 | 2.8 |
| 7 | E235N | 5.5 | 2.6 |
| 8 | E235S | 7.2 | 2.8 |
| 9 | E235A | 3.6 | 1.1 |
| 10 | D279A | 3.2 | 2.8 |
| 11 | D279N | 2.7 | 2.6 |
| 12 | D279Q | 7.1 | 2.6 |
| 13 | D279E | 6.1 | 2.6 |
| 14 | D279S | 3.2 | 1.3 |
| 15 | Q303L | 6.1 | 2.8 |
| 16 | Q303N | 5.9 | 2.8 |
| 17 | Q303A | 5.7 | 2.6 |
| 18 | Q303S | 3.7 | 1.1 |
| 19 | Y305F | 6.8 | 2.6 |
| 20 | Y305V | 6.3 | 2.6 |
| 21 | Y305Q | 6.5 | 2.6 |
| 22 | Y305N | 6.9 | 2.6 |
| 23 | Y305S | 4.2 | 1.1 |
| 24 | Y348F | 6.4 | 2.6 |
| 25 | Y348H | 7.0 | 2.8 |
| 26 | E350Q | 7.1 | 2.8 |
| 27 | E350D | 6.1 | 2.6 |
| 28 | E350V | 6.4 | 2.8 |
| 29 | Y402F | 5.6 | 2.8 |
| 30 | D233Q/E235Q | 3.8 | 2.8 |
| 31 | D233Q/E235D | 4.7 | 2.6 |
| 32 | D233Q/E235N | 5.0 | 2.8 |
| 33 | D233Q/E235S | 4.1 | 2.6 |
| 34 | D233Q/D279A | 4.8 | 2.6 |
| 35 | D233Q/D279N | 3.7 | 2.6 |
| 36 | D233Q/D279Q | 3.0 | 2.8 |
| 37 | D233Q/D279E | 3.0 | 2.8 |
| 38 | D233Q/Q303L | 4.3 | 2.6 |
| 39 | D233Q/Q303N | 4.8 | 2.8 |
| 40 | D233Q/Q303A | 4.6 | 2.8 |
| 41 | D233Q/Y305F | 4.5 | 2.8 |
| 42 | D233Q/Y305V | 4.1 | 2.6 |
| 43 | D233Q/Y305Q | 4.4 | 2.8 |
| 44 | D233Q/Y305N | 4.0 | 2.8 |
| 45 | D233Q/Y348F | 3.6 | 2.8 |
| 46 | D233Q/Y348H | 3.7 | 2.6 |
| 47 | D233Q/E350Q | 4.6 | 2.6 |
| 48 | D233Q/E350D | 4.2 | 2.6 |
| 49 | D233Q/E350V | 3.6 | 2.8 |
| 50 | D233Q/Y402F | 4.4 | 2.8 |
| 51 | Y282R | 5.3 | 1.0 |
| 52 | M283R | 3.1 | 0.2 |
| 53 | Q308R | 4.2 | 0.8 |
| 54 | E315R | 3.9 | 1.1 |
| 55 | E322R | 3.8 | 0.6 |
| 56 | H236A/D237A | 4.6 | 0.9 |
| 57 | M283A/K286A | 5.1 | 0.1 |
| 58 | T281A | 4.1 | 1.0 |
| 59 | E315A/R320A/E322A/K323A | 3.5 | 1.0 |
| 60 | E833A | 4.7 | 0.2 |
| 61 | D233Q/Y348L | 3.6 | 0.7 |
| 62 | D233Q/Y348R | 4.2 | 0.1 |
| 63 | D233Q/E350A | 3.8 | 0.8 |
| 64 | D233Q/E350N | 2.7 | 0.8 |
| 65 | D233Q/D405A | 7.0 | 0.1 |
| 66 | D233Q/D405N | 6.1 | 0.1 |
| 67 | D233Q/R406A | 5.6 | 0.9 |
| 68 | D233Q/R406Q | 4.0 | 0.5 |
| 69 | P184Q/D233Q | 3.3 | 0.9 |
| 70 | D231Q/D233Q | 6.0 | 0.6 |
| 71 | D233Q/D279S | 6.4 | 0.2 |
| 72 | D233Q/D279H | 3.8 | 0.3 |
| 73 | D233Q/T281F | 6.1 | 0.1 |
| 74 | D233Q/Q303S | 5.1 | 0.1 |
| 75 | D233Q/Q303F | 4.4 | 0.8 |
| 76 | D233Q/Y305S | 3.9 | 1.0 |
| 77 | D233Q/Y305H | 4.1 | 0.7 |
| 78 | D233Q/Y305D | 5.8 | 0.7 |
| 79 | D233Q/Y402W | 3.9 | 0.7 |
| 80 | D233Q/Y402H | 3.7 | 0.4 |
| 81 | D233Q/Y402L | 2.9 | 0.6 |
| 82 | D233Q/W803A | 4.4 | 0.7 |
| 83 | D233Q/Q303L/E350D | 5.4 | 0.1 |
| 84 | R119A/D233Q | 2.9 | 0.3 |
| 85 | R119N/D233Q | 6.2 | 0.1 |
| 86 | R119E/D233Q | 5.9 | 0.1 |
| 87 | H122A/D233Q | 5.0 | 0.1 |
| 88 | H122F/D233Q | 7.8 | 0.1 |
| 89 | R186N/D233Q | 5.2 | 0.1 |
| 90 | F187A/D233Q | 4.2 | 0.4 |
| 91 | N193A/D233Q | 5.4 | 0.1 |
| 92 | D233Q/Q303V | 2.0 | 0.2 |
| 93 | D233Q/Q303I | 4.3 | 0.1 |
| 94 | W185R | 5.3 | 0.1 |
| 95 | V251R | 6.2 | 0.1 |
| 96 | V317R | 4.7 | 0.1 |
| 97 | D233Q/Y348H/D405A | 2.1 | 1.9 |
| 98 | D233Q/Y348H/R406A | 2.3 | 1.6 |
| 99 | D233Q/E350A/D405A | 3.8 | 0.4 |
| 100 | D233Q/E350A/R406A | 2.3 | 1.0 |
| 101 | D233Q/Y348H/E350A | 2.7 | 1.6 |
| 102 | D233Q/Y348H/E350A/D405A | 2.6 | 1.6 |
| 103 | D233Q/D279Q/Q303L | 2.6 | 1.6 |
| 104 | D233Q/D279Q/Y402F | 2.5 | 1.3 |
| 105 | D233Q/Q303L/Y402F | 2.9 | 1.2 |
| 106 | D233Q/D279Q/Q303L/Y402F | 3.6 | 0.3 |
| 107 | D233Q/D279Q/Y348H | 3.4 | 0.2 |
| 108 | D233Q/D279Q/E350A | 3.1 | 1.1 |
| 109 | D233Q/D279Q/D405A | 6.5 | 0.4 |
| 110 | D233Q/Q303L/Y348H | 2.8 | 1.1 |
| 111 | D233Q/Q303L/E350A | 2.1 | 1.3 |
| 112 | D233Q/Q303L/E350Q | 4.0 | 0.8 |
| 113 | D233Q/Q303L/D405A | 5.9 | 0.5 |
| 114 | D233Q/Y348H/Y402F | 3.9 | 0.9 |
| 115 | D233Q/E350A/Y402F | 3.9 | 0.8 |
| 116 | D233Q/Y402F/D405A | 3.3 | 1.1 |
| 117 | D233Q/Y282R/D405A | 2.5 | 0.4 |
| 118 | D233Q/Y282R/R406A | 3.5 | 0.4 |
| 119 | D233Q/D279Q/Y282R | 3.3 | 0.4 |
| 120 | D233Q/Y282R/Y402F | 2.9 | 0.4 |
| 121 | D233Q/Y282R/Y402W | 2.2 | 0.6 |
| 122 | D233Q/Y282R/Q303L/E350D | 2.8 | 1.2 |
| 123 | D233Q/Y282R/Q303L/E350A | 3.9 | 0.5 |
| 124 | D233Q/Y282R | 4.3 | 0.5 |
| 125 | D233Q/Y282R/Q303L | 3.5 | 0.5 |
