# Supplementary material for: Generation of efficient mutants of endoglycosidase from Streptococcus pyogenes and their application in a novel one-pot transglycosylation reaction for antibody modification
Source: PLoS One. 2018 Feb 23;13(2):e0193534. doi: 10.1371/journal.pone.0193534 (PMC5825150; doi:10.1371/journal.pone.0193534)
Supplement: S2 Table — The hydrolysis and transglycosylation efficiencies of Endo-S D233Q and other mutants that displayed reduced hydrolysis or enhanced transglycosylation efficiency at any time point compared to the Endo-S D233Q mutant. (DOCX) [file pone.0193534.s002.docx]

|  | % hydrolysis | | | | | | % transglycosylation | | | | | |
| --- | --- | --- | --- | --- | --- | --- | --- | --- | --- | --- | --- | --- |
| Mutant | 1h | 2h | 4h | 8h | 24h | 48h | 1h | 2h | 4h | 8h | 24h | 48h |
| D233Q | 0 | 11 | 17 | 30 | 55 | 71 | 52 | 83 | 82 | 80 | 75 | 66 |
| H122A/D233Q | 0 | 0 | 21 | 26 | 45 | 54 | 76 | 84 | 85 | 81 | 78 | 71 |
| H122F/D233Q | 0 | 0 | 0 | 0 | 0 | 0 | 3 | 8 | 13 | 28 | 52 | 60 |
| F187A/D233Q | 0 | 0 | 23 | 32 | 55 | 72 | 63 | 82 | 82 | 77 | 76 | 70 |
| D233Q/D279S | 0 | 0 | 0 | 0 | 0 | 19 | 21 | 35 | 50 | 64 | 66 | 66 |
| D233Q/D279Q | 0 | 0 | 0 | 7 | 22 | 30 | 47 | 63 | 77 | 78 | 78 | 74 |
| D233Q/Q303L | 0 | 0 | 12 | 20 | 34 | 42 | 33 | 51 | 79 | 94 | 96 | 91 |
| D233Q/Y348H | 0 | 0 | 16 | 22 | 35 | 48 | 53 | 63 | 72 | 75 | 74 | 73 |
| D233Q/E350A | 0 | 0 | 0 | 12 | 28 | 32 | 48 | 69 | 86 | 91 | 89 | 87 |
| D233Q/E350N | 0 | 0 | 0 | 13 | 27 | 37 | 52 | 77 | 89 | 91 | 85 | 84 |
| D233Q/E350Q | 0 | 18 | 24 | 28 | 45 | 53 | 75 | 92 | 91 | 90 | 82 | 74 |
| D233Q/E350D | 0 | 0 | 0 | 12 | 25 | 32 | 49 | 66 | 82 | 89 | 89 | 88 |
| D233Q/Y402F | 0 | 0 | 0 | 0 | 0 | 15 | 36 | 49 | 66 | 77 | 82 | 82 |
| D233Q/D405A | 0 | 0 | 11 | 19 | 34 | 48 | 78 | 90 | 92 | 92 | 85 | 83 |
| D233Q/R406Q | 0 | 0 | 0 | 0 | 0 | 0 | 8 | 14 | 23 | 33 | 48 | 53 |
| D233Q/Y282R/D405A | 0 | 0 | 0 | 0 | 0 | 0 | 16 | 25 | 41 | 56 | 66 | 71 |
| D233Q/Q303L/E350A | 0 | 0 | 0 | 0 | 0 | 0 | 5 | 9 | 13 | 24 | 42 | 49 |
| D233Q/Q303L/E350Q | 0 | 0 | 0 | 0 | 0 | 12 | 15 | 33 | 50 | 69 | 87 | 92 |
| D233Q/Q303L/E350D | 0 | 0 | 0 | 0 | 0 | 0 | 5 | 12 | 21 | 34 | 58 | 66 |
| D233Q/Q303L/Y402F | 0 | 0 | 0 | 0 | 0 | 0 | 5 | 9 | 15 | 27 | 44 | 52 |
| D233Q/Q303L/D405A | 0 | 0 | 0 | 0 | 0 | 0 | 10 | 19 | 30 | 43 | 63 | 72 |
| D233Q/Y402F/D405A | 0 | 0 | 0 | 0 | 0 | 0 | 12 | 22 | 39 | 55 | 72 | 77 |
