# Supplementary material for: Generation of efficient mutants of endoglycosidase from Streptococcus pyogenes and their application in a novel one-pot transglycosylation reaction for antibody modification
Source: PLoS One. 2018 Feb 23;13(2):e0193534. doi: 10.1371/journal.pone.0193534 (PMC5825150; doi:10.1371/journal.pone.0193534)
Supplement: S3 Table — The one-pot transglycosylation efficiency by Endo-S D233Q and Endo-M N175Q using various concentrations of SGP as a donor substrate. (DOCX) [file pone.0193534.s003.docx]

|  | % transglycosylation | | | | |
| --- | --- | --- | --- | --- | --- |
| SGP:Antibody | 2h | 4h | 8h | 24h | 48h |
| 50:1 | 47 | 65 | 80 | 84 | 78 |
| 100:1 | 67 | 80 | 89 | 87 | 86 |
| 200:1 | 90 | 92 | 93 | 91 | 88 |
| 300:1 | 92 | 95 | 95 | 93 | 91 |
| 400:1 | 93 | 95 | 95 | 95 | 92 |
