# Supplementary figures and images for: Generation of efficient mutants of endoglycosidase from Streptococcus pyogenes and their application in a novel one-pot transglycosylation reaction for antibody modification
Source: PLoS One. 2018 Feb 23;13(2):e0193534. doi: 10.1371/journal.pone.0193534 (PMC5825150; doi:10.1371/journal.pone.0193534)

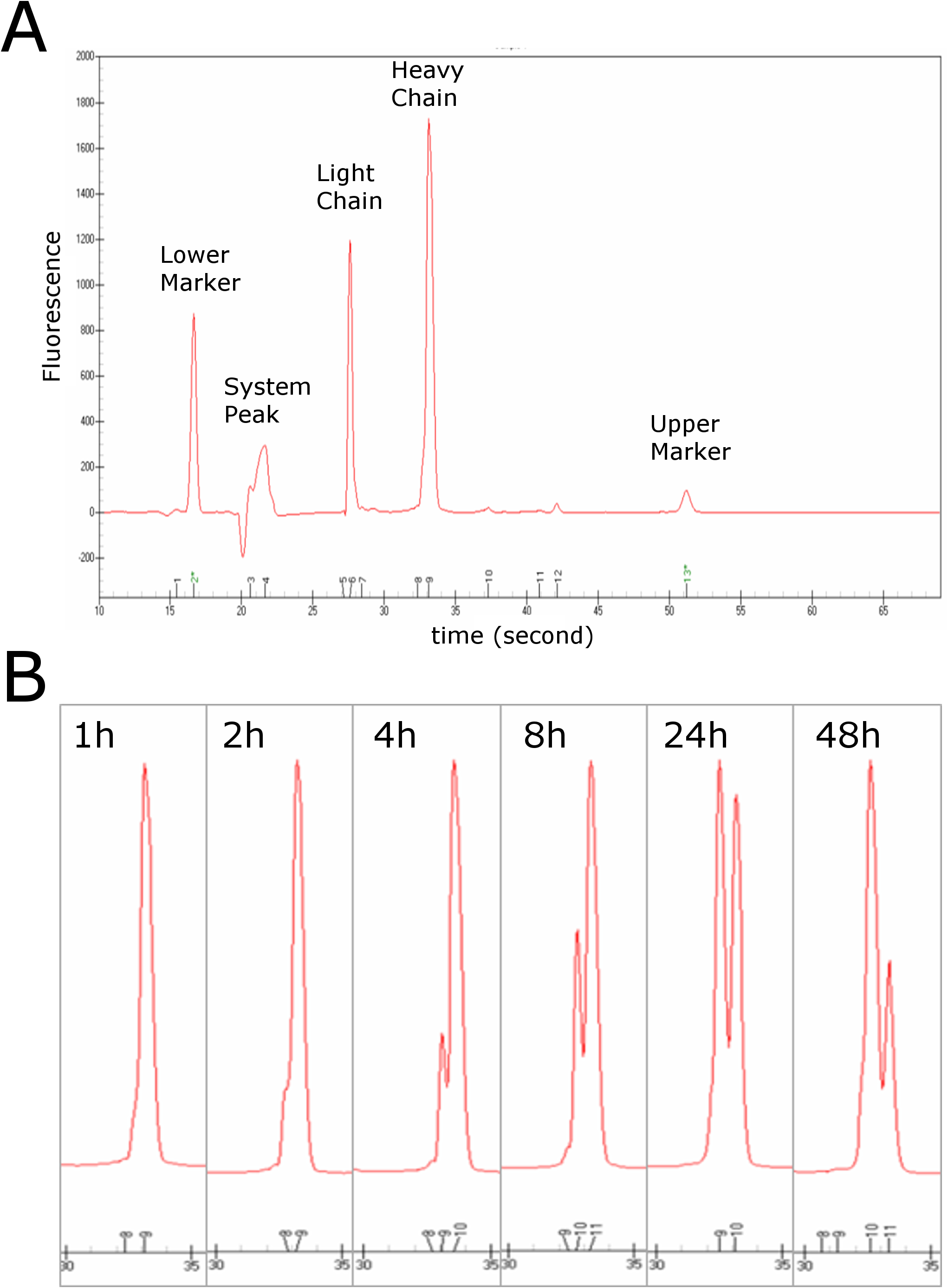

Supplement: S1 Fig — (A) The electropherogram of the Endo-S D233Q-catalyzed deglycosylation reaction at 1 h obtained using Experion Automated Electrophoresis Station (Bio-Rad). (B) Comparison of the heavy chain peak at various time points of the deglycosylation reaction demonstrates that the relative amount of glycosylated and deglycosylated heavy chains can be approximately quantified. (TIF) [file pone.0193534.s004.tif]
